# Home hemodialysis technique survival: insights and challenges

**DOI:** 10.1186/s12882-023-03264-5

**Published:** 2023-07-11

**Authors:** Estelle Tran, Oliver Karadjian, Christopher T. Chan, Emilie Trinh

**Affiliations:** 1 Department of Medicine, Royal College of Surgeons in Ireland, Dublin, Ireland; 2 Division of Nephrology, Department of Medicine, McGill University Health Center, 1650 Av Cedar, L4-510, Montreal, QC H3G 1A4 Canada; 3 Division of Nephrology, University Health Network, Toronto, ON Canada

**Keywords:** Home hemodialysis, Technique failure, Training failure, Technique survival, Discontinuation

## Abstract

Home hemodialysis (HHD) offers several clinical, quality of life and cost-saving benefits for patients with end-stage kidney disease. While uptake of this modality has increased in recent years, its prevalence remains low and high rates of discontinuation remain a challenge. This comprehensive narrative review aims to better understand what is currently known about technique survival in HHD patients, elucidate the clinical factors that contribute to attrition and expand on possible strategies to prevent discontinuation. With increasing efforts to encourage home modalities, it is imperative to better understand technique survival and find strategies to help maintain patients on the home therapy of their choosing. It is crucial to better target high-risk patients, examine ideal training practices and identify practices that are potentially modifiable to improve technique survival.

## Introduction

Home dialysis modalities, including home hemodialysis (HHD) and peritoneal dialysis (PD), offer several benefits for patients with end-stage kidney disease (ESKD). HHD is associated with improvements in blood pressure, abnormalities of mineral metabolism, sleep quality and regression of left ventricular hypertrophy [1–4] HHD encourages patient autonomy by allowing patients to direct their own treatments as well as flexibility to adjust their dialysis schedule while avoiding time and cost of frequent travel to a dialysis center. Moreover, several studies have demonstrated quality of life benefits with HHD as well as significant lower costs compared to conventional in-center hemodialysis [3, 5, 6]. Nonetheless, despite demonstrated advantages, the worldwide prevalence of HHD remains low with large variation in uptake—approximately 18% of all dialysis patients in New Zealand, 9% in Australia, 3–6% in Canada [3] and 2% in the United States [7, 8]. With ongoing efforts to increase uptake of home dialysis therapies, the innovation of dialysis technologies, different types of dialysis regimen to accommodate patients’ needs and additional centers offering this modality, the use of HHD has increased in many areas of the world. Furthermore, in the context of the COVID-19 pandemic, there has been even further advocacy worldwide to increase uptake of home therapies to reduce potential exposures. However, high attrition rates remain a challenge. Little is known on what patient or center-specific characteristics predict discontinuation and what factors are potentially modifiable. In this review, we aim to elucidate on what is currently known about technique survival in HHD and its challenges.

### Challenges in defining technique failure

Technique failure (TF) is defined as a transfer to an alternative dialysis modality for a predetermined amount of time. Some studies also include death and renal transplantation as reasons for therapy discontinuation [9–12]. In the case of HHD, PD and in-center hemodialysis are considered the two main alternative modalities. It remains unknown what optimal time period most accurately defines TF or is associated with adverse outcomes. In fact, studies have employed a variety of definitions including 30, 45, 60, 90, and 180-days. Transfers may often be temporary in the context of an acute illness or hospitalization, and as such, too short a time frame used for the definition of TF may not accurately represent true discontinuation as patients may eventually return to their initial modality [13]. Conversely, using a longer time frame may lead to patients getting lost to follow-up or developing other unrelated complications. As it stands, there is a lack of homogeneity and standardization in the literature in defining TF which renders it difficult to accurately determine implications on clinical outcomes and compare studies. As a comparison, Clarke et al*.* investigated 10 Canadian dialysis programs with patients on PD and concluded that the approach used to report TF can have a major impact on the reported risk of TF. The use of different time windows for observation for a return to PD after switching to HD resulted in a difference of 16% in risks of TF. They also found that 90% of patients who switched modality returned to PD within 180 days [14]. A 30-day definition allowed to capture acute intercurrent illness contributing to increased morbidity and mortality while a 180-day definition provided a timeframe where it was unlikely the patient would return to PD. As such, the authors emphasized that different definitions addressed distinct clinical aspects. This study focused primarily on PD; hence, its conclusions should be carefully examined when directed to HHD. It is evident that the lack of standardization in defining TF in HHD makes it difficult to compare studies and identify modifiable practices. Therefore, with the current literature available, it is important to acknowledge this limitation before drawing meaningful conclusions.

### Rates of technique failure in home hemodialysis

Discontinuation rates in HHD vary considerably in the literature with 1-year rates between 2 and 30%, largely owing to different patient populations and definitions being used [12, 15–17]. A study by Perl et al*.* [9]*,* reported rates during the first 6 months of HHD initiation of 7% between 2003–2007 and 14% between 2008–2012. Indeed, they found that discontinuation rates increased over the years and that they were 2.5-fold higher in 2008–2012 compared to the earlier period of 2003–2007. Conversely, a cohort study by Seshasai et al. [18] analyzed data from a dialysis provider in the United States and reported a much higher attrition rate of 24.9% in the first year, but included transplantation and death in their definition. In addition, a retrospective study by Choo et al*.* [15] including patients enrolled in an Australian nocturnal HHD program determined that TF was observed in 30% of patients (33 out of 109 patients—16 were transferred to in-center hemodialysis, 1 to PD and 16 died).

Finally, several studies [9, 13, 18] have demonstrated that discontinuation rates are not constant over time and appear to be highest during the first year followed by a significant decrease over time. Trinh et al*.* [13] analyzed patients from the Canadian Organ Replacement Registry and reported rates of TF in HHD patients of respectively 22% at year 1, 8% at year 2, 11% at year 3 and 6% at year 4. Perhaps, the technical complexity of HHD including self-cannulation, machine set-up and the need of adjustments of the hemodialysis parameters may explain the initial elevated attrition rates and these rates likely decrease subsequently as patients are well-established on their treatment. These variations in discontinuation rates are likely related to center-specific practice patterns, evolving patient characteristics and potentially changing selection criteria over time with patients with more comorbidities being treated with HHD.

### Risk factors—patient- related factors

**Fig. 1:**
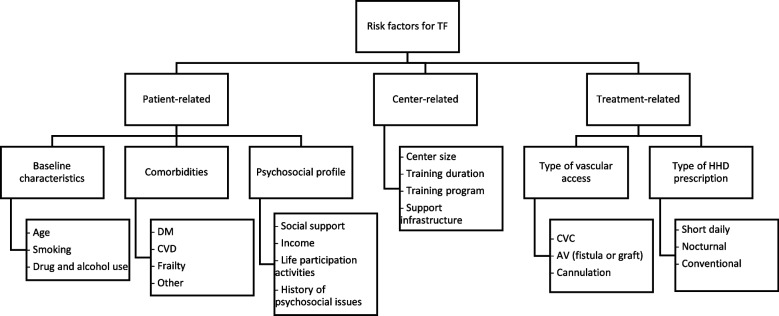
Risk Factors for HHD TF. HHD, home hemodialysis; TF, technique failure; DM, diabetes mellitus; CVD, cardiovascular disease; CVC, central venous catheter; AV, arteriovenous

Patient characteristics contribute to risk of TF in HHD (Fig. 1). Studies have identified the following characteristics as risk factors: older age, cardiac disease, diabetes, drug use, alcohol, and smoking [9, 10, 16, 18]. In fact, diabetic patients tend to experience more vascular access difficulties and medical interventions whereas patients above the age of 65 and those new to dialysis were two times more likely to discontinue treatment as compared to younger patients [9, 18]. Paterson et al*.* [10] confirmed that a history of coronary artery disease (CAD) and diabetes were also risk factors for TF in HHD. Conversely, ESKD from renovascular or polycystic kidney disease was associated with a lower risk of TF than from diabetes. In addition, a retrospective study by Seshasai et al. [18] of adult HHD patients in the United States from 2007–2009 demonstrated that patients listed for kidney transplant were 27% less likely to discontinue HHD treatment. This likely reflects a younger and less comorbid patient population. In fact, Schachter et al*.* [19] investigated the impact of patients’ frailty on TF and concluded that a higher Clinical Frailty Scale (CFS) was associated with a higher risk of TF.

Furthermore, there also appears to be some racial differences in the risk of TF [20, 21]. Mehrotra et al*.* [22] examined racial disparities in home dialysis modalities in the United States. Among patients who were treated with HHD, blacks were the only ethnic minority that had at a higher risk for transfer to in-center HD compared to their white counterparts. However, blacks had a lower mortality rate than whites. Trinh et al*.* [23] also studied the association between race and TF among Canadian HHD patients between 1996–2012; they did not observe any significant association between race and TF.

### Risk-factors – center specific factors

Some center-specific factors have been shown to be associated with HHD discontinuation. Trinh et al*.* [13] reported that small facility size was a predictive factor for TF in HHD. Perhaps this could be explained by larger centers having more experience with patients on HHD and more resources and staff to support patients and their caregivers [24]. Similarly, it has been shown that facilities with a higher proportion of patients initiating home hemodialysis treatment as initial dialysis modality also seem to have lower risks of TF [25].

More recently, a study by Morin et al*.* [26] showed a correlation between duration of training and rates of technique failure. They found that a longer training period was associated with TF. Recent studies suggest that training programs that engage both patient and patient’s family, actively participating in the pre-dialysis and pre-ESKD classes can yield lower rates of TF [27–29]. This confirms the importance of center experience and future studies should aim to elucidate modifiable center and training practices that can help improve technique survival.

### Risk factors—treatment-related factors

Several treatment-related factors can contribute to an increased discontinuation risk in HHD patients. The type of vascular access seems to have an impact; an observational cohort study by Perl et al*.* [30] compared HHD patients using a central venous catheter (CVC) versus a self-cannulated arteriovenous (AV) fistula or graft. They noted that CVC use was associated with a higher risk of TF. Furthermore, buttonhole cannulation (BH) is another type of vascular access intervention that has been widely used in HHD because it may make cannulation easier for patients. However, this technique can be associated with risks of local and systemic infections which may potentially lead to subsequent discontinuation [31]. Nesrallah et al*.* [32] observed in their study that out of the 56 patients who were on nocturnal HHD, 10 experienced a bacterial infection where patients exited the program or switched modality. On the other hand, a randomized single-center trial by Vaux et al*.* did not observe an increase in bacteremia events or bleeding time with BH which was shown to be associated with fewer access interventions [33].

In addition, there has been controversy on whether the type of HHD prescription itself is associated with TF. On one hand, some studies have shown that nocturnal HHD seems to be associated with an increased risk of TF due to high risks of access failure as compared to conventional HHD [34] with 3 times greater risks of a septic event. Jun et al*.* [35] recruited patients from 6 Australian centers who were performing extended-hours home hemodialysis and found that higher frequency hemodialysis was also associated with a higher risk of TF and death. Conversely, Tennankore et al*. * [36] compared patients receiving short daily HHD, nocturnal HHD and conventional HHD in Canada and showed no significant differences in risk of TF. Therefore, it is still unclear whether the HHD prescription itself modifies technique survival.

### Causes of discontinuation

Both psychosocial and medical reasons may lead to discontinuation [16, 37, 38]. Shah et al*.* reported that out of the 23 patients who experienced TF, medical instability was the predominant reason for modality change (65%), followed by patient or caregiver burnout (13% and 6%) and patient choice (9%) [38]. The study also mentioned that the multidisciplinary team flagged 22 patients as “vulnerable for HHD failure” prior to training and more than half of them experienced TF or death. Similarly, Jayanti et al*.* followed 166 patients from the Greater Manchester East Sector Renal Network and over a period of 8 years, 24 patients switched modality during follow-up and the main reasons included medical issues, lack of motivation, lack of confidence and inability to cope with the stress and old age [16]. Patients also reported that family dynamics, interference with life at home, time constraints and lack of carer support can also make it difficult to continue with the treatment. Komenda et al*.* described the 2-year outcomes of a provincial HHD program in British Columbia (Canada) and reported that the main reasons for discontinuation were inadequate social support, unspecified medical reasons and dialysis withdrawal which are similar to the ones stated in the previously mentioned studies [39]. This only further consolidates the importance of understanding patients’ psychosocial needs prior to initiation of HHD and prioritize a support infrastructure to help deal with these issues. Another study conducted by Pauly et al. included 247 NHD patients of the Canadian Slow Long nightly ExtEnded dialysis Programs (CAN-SLEEP) from 1994 to 2006 and concluded that 14.6% of the cohort experienced TF due to adverse events, mainly vascular access complications [40]. Although many studies have focussed on the medical and psychological determinants of technique survival, we also want to recognize that others have also included patient’s non-adherence such as skipping treatments and patient’s relocation as potential reasons for discontinuation of HHD [10, 12, 41].

Furthermore, causes of HHD discontinuation appear to vary over time. Paterson et al*.* reported that TF due to psychosocial reasons appeared earlier with a median time of 8.9 months while TF due to medical reasons and safety concerns appeared later into the treatment with a median time of 26 and 19 months respectively [10]. Comparatively, a study by Pauly et al. [11] demonstrated that older age and frailty were reasons for early discontinuation. Interestingly, patients of higher economic status and countries with longer training times appear to experience lower TF rates [11]. Later causes of discontinuation have been reported to include patient burnout, change in physical or cognitive capacity, access to dialysis facilities, and complications with dialysis machines [9]. Therefore, it is crucial to recognize that reasons for discontinuation may be time sensitive so that healthcare teams may develop more targeted strategies to identify patients at high risk of TF and to implement timely interventions to prevent discontinuation.

It is important to acknowledge that many of the studies examining technique survival in HHD are observational in nature and thus, subject to the inherent biases of observational studies. Some other limitations that need to be emphasized are: different study populations, distinct dialysis prescriptions (including the dose, the frequency, the time of delivery), and different types of dialysis technology. Therefore, these limitations should be considered when interpreting the current state of the literature.

### Comparison with peritoneal dialysis

**Fig. 2:**
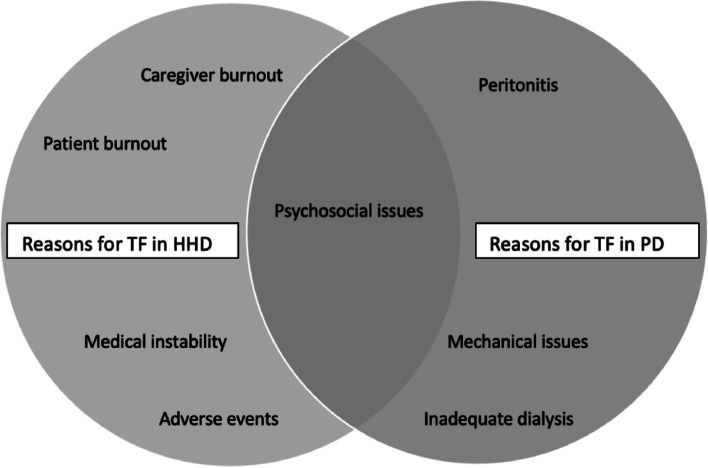
Comparison between the main reasons for technique failure in HHD versus in PD. TF, technique failure; HHD, home hemodialysis; PD, peritoneal dialysis

In comparison with HHD, rates of technique failure in PD vary between 4.9% and 26.2% in the literature also with higher rates during the first year of initiation of dialysis therapy [42–44]. However, studies that have directly compared these two modalities reported a significant higher risk for TF in PD compared to HHD. A Canadian multicenter study noted a 50% risk reduction in TF in HHD compared to PD while a US matched cohort study reported a 37% lower risk for TF relative to PD [45, 46]. Furthermore, Trinh e*t al*. [13] compared technique failure in both modalities and demonstrated that while the main cause of discontinuation in HHD was psychosocial issues, the majority of PD patients experienced treatment failure due to medical issues (Fig. 2).

Risk factors for TF appear to differ between PD and HHD. Similar risk factors include: a higher degree of comorbidity, diabetes, smoking, cardiovascular disease, and smaller center size. In contrast, age has a variable effect on technique survival between modalities. While older age has clearly been shown to be associated with higher risk of TF in HHD [12, 16, 47], the effect of age on technique failure is variably reported in PD. In some studies, older patients have been shown to have increased technique failure risk [43, 48] whereas other studies have shown a lower risk in patients > 65 years old [49]. This may be related to increasing utilization of assisted PD in older patients in some areas of the world which may be associated with a lower risk of TF [50].

### Complications associated with technique failure

HHD discontinuation usually leads to transition to conventional in-center hemodialysis. This transition period has been shown to be associated with poor outcomes and a higher risk of morbidity and mortality. In a study by Shah et al*.* that compared 60 patients who stayed on HHD or were transplanted with 23 patients who had to transition to facility-based HD*,* the 90-day mortality in patients who experienced HHD technique failure was significantly higher compared to patients who remained on HHD [38]. Similarly, Nitsch et al*.* identified patients on HHD from the UK Renal Registry (UKRR) database and revealed that out of 130 patients who decided to stop HHD, 19 deaths were recorded within 3 months after switching to an alternative modality [51]. Semple et al*.* noted a similar observation in patients from the Australia and New Zealand Dialysis and Transplant Registry; HHD treatment failure was associated with an increased mortality compared to continued HHD in both early (< 90 days) and later periods (> 90 days) after treatment failure [52]. Without doubt, the transition period from one renal replacement therapy to another is a particularly vulnerable time, both in context of the acute medical issues leading to TF, but also due to the unplanned nature of the transition. Adequate evaluation of patient’s medical and psychosocial needs is essential [29]. It is imperative to identify high risk patients early on and to target them in order to not only prevent TF, but also to ensure a better and smoother transition between renal replacement modalities.

### Prioritizing home hemodialysis during a pandemic

During the era of a pandemic, patients undergoing renal replacement therapy are particularly vulnerable; they are at increased risk of exposure and complications from SARS-CoV-2 due to frequent hospital visits in addition to underlying comorbidities and an immunocompromised state [53–56]. In addition, the need for isolation and fear of infection may create a psychological burden for these patients. In this context, home dialysis modalities are an even more appealing alternative [57]. With the combination of telemedicine and current dialysis technologies, HHD uptake may be facilitated with close virtual monitoring of patients, training and education of healthcare practitioners, staff, and patients [58–60]. In fact, the predominant use of telehealth in HHD patients during the Covid-19 pandemic has shown to be a positive addition to standard HHD regimen by allowing remote contact between patients and physicians as well as promoting a stronger patient-healthcare professional relationship [59, 60]. By addressing factors associated with TF and complications in HHD, this at-home infrastructure can be appropriately promoted to targeted patients who would highly benefit from this modality during and after the pandemic [61].

### Strategies for prevention of technique failure in the current era

With increasing efforts to encourage uptake of home dialysis modality in the current era, the importance to improve technique survival is primordial. While most of the literature focuses on increasing uptake of HHD, little is known on strategies to prevent discontinuation. It is crucial to better understand causes and clinical factors associated with technique survival to prevent discontinuation and better support patients on HHD.

To improve technique survival, several strategies and interventions can be considered (Table 1). First, it is important to focus on building a comprehensive and well-established training program. This requires extensive physician and patient training, staff and nurse education, and infrastructure support. It is essential that patients are informed about the benefits, but also potential risks of HHD. Indeed, a cross-sectional survey conducted by McLaughlin et al. revealed that many patients who undergo HHD feel unprepared due to a lack of education [28]. Training programs that take the time to properly train their patients to master this skill can give them the confidence they need to continue their treatment at home [62]. For instance, fear of self-cannulation is a significant reason for discontinuation of this therapy in some self-care patients [3, 10, 18, 63]. Hence, a facility equipped with experienced staff dedicated to HHD, a complete multidisciplinary staff including social workers, dieticians, nurses, and physicians who offer maximum support to the patient and their caregivers are all factors that may contribute to a lower risk of treatment failure. It is crucial that we emphasize on developing a program that focuses on a patient-centered approach by, first and foremost, addressing patients’ concerns and priorities.

**Table 1:** Strategies to Prevent Discontinuation in Home Hemodialysis

Problem	Strategy
Lack of patient education/ training	Build a comprehensive training program with extensive physician, nurse, staff, and patient support
Lack of physician expertise	Increase in exposure to home hemodialysis during nephrology training
Lack of confidence/anxiety	Identify and address patient’s psychosocial needs early on, individualized education, routine psychosocial support, peer support network, and consider virtual monitoring
Patient burnout	Routine psychosocial assessments with targeted support Support groups Consider care partner or paid helper
Care partner burnout	Support groups Consider respite care
Early discontinuation of vulnerable patients	Create a robust pre-dialysis screening of patients, and target patients who will need extra support and close monitoring
Technical complexities	Offer adequate individualized patient training and support Support groups

In addition, there seems to be a gap in physician education on HHD as evidence demonstrates that there is a lack of time spent on HHD exposure for nephrology fellows [64, 65]. A survey completed by Australian nephrologists confirmed that the reluctance to expand home hemodialysis therapies are due to a lack of nephrologist expertise in HHD as well as sufficient physical infrastructure for adequate training [66]. Moreover, it was also revealed that only 16% felt prepared and well-trained for HHD [67]. An increase in exposure to home hemodialysis during nephrology fellowship is needed to improve confidence in the use of this modality.

Many HHD patients express psychosocial concerns which are often underestimated by the medical multidisciplinary team. This aspect can influence the patient’s endurance on HHD and lead to patient and/or caregiver burnout. Indeed, in a cross-sectional analysis by Suri et al*.* which enrolled patients in the Frequent Hemodialysis network (FHN), over 50% of patients on HHD who completed the questionnaire believed that their caregivers were overextended, and this self-perceived burden had a significant association with a deterioration in quality of life [68]. Furthermore, the need to self-adjust dialysis parameters might also contribute to patient stress and anxiety [63]. Patients may also have concerns about changes in their daily schedule due to the HHD treatment. They might have to adjust their usual activities, hobbies, employment, social engagements which could have an impact on their mental health. This only emphasizes the importance of implementation of a program tailored to the patients’ and care partners’ priorities. For instance, a pre-dialysis discussion with a multidisciplinary team to address the patient’s concerns in early stages as well as a continuous assessment of patient’s psychosocial needs should be encouraged [1, 69, 70]. Involving an experienced psychologist or social worker early on could benefit in detecting and addressing psychosocial stressors. In addition, as home dialysis therapy can become very isolating, promoting a peer support network can create a feeling of community amongst HHD patients and their care partners such as a local group support and/or web-based support. Opportunities to speak with other HHD patients may help give patients extra confidence in pursuing or continuing their home therapy [71]. Without doubt, continuous reassurance and providing adequate resources to support patients must be practiced throughout the process including offering additional training or assistance when necessary.

Some patients may require the help of a care partner to perform home dialysis. In these cases, the success of patients on HHD may be highly impacted by their relationship with their caregiver. Therefore, primary caregivers should be well-informed of the potential challenges that come with this responsibility and a well-established support infrastructure in place to be able to aid in providing appropriate assistance should issues occur. The availability of intermittent respite care should be considered to allow the patient and/or caregiver some time-off and prevent burnout. Another option to consider is a paid helper if the patient can financially support this alternative [1, 37, 72].

Finally, one of the most crucial strategies to prevent discontinuation in HHD is to identify vulnerable patients who are the most at-risk [18, 73]. With a robust screening of patients, we can target the ones who will need extra support and close monitoring. Furthermore, perhaps a more comprehensive selection process should be considered in certain situations to avoid premature treatment failure and associated complications. Fortunately, with the present increase in the appeal of home dialysis modalities, there is a remarkable shift in policy change to expand its adoption. Multiple strategies and technological advancement to ease the use of HHD are being developed which may help improve the sustainability of hemodialysis at home. Moreover, the growth of telehealth technologies may also help promote and assist patients on home dialysis.

### Further direction

To better ensure success in HHD, programs in dialysis centers should implement discontinuation rates as a performance indicator and locally explore quality improvements initiatives to examine and improve technique survival. Further research is needed to expand on the best strategies to improve technique survival among patients on HHD. Additional qualitative studies that focus on patients’ and caregivers’ perspective are crucial for a better understanding of factors that lead to discontinuation, what support structures are needed and what policy changes are required to improve technique survival. In addition, larger scale international prospective collaborations are needed to standardize technique survival definitions and help identify modifiable practices, especially center-specific factors, that can improve technique survival in HHD. This could be achieved through an international prospective study focused on HHD, similar to the Peritoneal Dialysis Outcomes and Practice Patterns Study (PDOPPS) [74]. A better understanding of technique survival is crucial to increase home hemodialysis uptake. In ideal settings, based on worldwide experiences, we believe home hemodialysis can be achieved in 5–15% of all prevalent dialysis patients provided a fair and unbiased discussion of the options is presented to patients and their care partners [75].

## Conclusion

With increasing efforts to encourage home modalities, it is imperative to better understand technique survival and find strategies to help maintain patients on the home therapy of their choosing. It is crucial to better target high-risk patients, examine ideal training practices and identify center-specific practices that are potentially modifiable to improve technique survival.

## Data Availability

Not applicable.

## Abbreviations

AV: Arteriovenous
BH: Buttonhole
CAD: Coronary Artery Disease
CFS: Clinical Frailty Scale
CVC: Central Venous Catheter
ESKD: End-Stage Kidney Disease
FHN: Frequent Hemodialysis Network
NHD: Nocturnal Home Hemodialysis
HD: Hemodialysis
HHD: Home Hemodialysis
PD: Peritoneal Dialysis
SARS-CoV-2: Severe Acute Respiratory Syndrome Coronavirus 2
TF: Technique failure
UKRR: United Kingdom Renal Registry
